# The Evolution of Professional Nursing Culture in Italy: Metaphors and Paradoxes

**DOI:** 10.1177/2333393614549372

**Published:** 2014-10-08

**Authors:** Gennaro Rocco, Dyanne D. Affonso, Linda J. Mayberry, Alessandro Stievano, Rosaria Alvaro, Laura Sabatino

**Affiliations:** 1 Center of Excellence for Nursing Scholarship, Rome, Italy; 2 New York University, New York, USA; 3 Tor Vergata University, Rome, Italy

**Keywords:** culture / cultural competence, Europe / Europeans, focus groups, health professionals, research, qualitative, nursing

## Abstract

We explored the perceptions of Italian nurses regarding their developing culture as a health profession. We sought to understand the ongoing evolution of the nursing profession and the changes that were central to it becoming an intellectual discipline on par with the other health professions in Italy. In 2010, the Regulatory Board of Nursing established a center of excellence to build evidence-based practice, advocate for interdisciplinary health care, and champion health profession reforms for nursing. In this study, focus groups—involving 66 nurse participants from various educational, clinical, and administrative backgrounds—were utilized to better ascertain how the profession has changed. Six themes, three of them metaphors—“vortex,” “leopard spots,” and “deductive jungle”—explain nurses’ experiences of professional change in Italy between 2001 and 2011 and the multiple dimensions that characterize their professional identity and autonomy.

Governments around the world have set out an agenda to realize an unprecedented shift in the delivery of health care. This transformation is motivated by the need to provide quality, affordable, and sustainable health care. National health care reform initiatives are focused on improving access to quality care, integrating services across the continuum of care, managing costs, and building the long-term sustainability of the health care system as a whole. Undergirding these paradigm shifts are efforts to understand the nature of professional culture and culture change in health care systems and organizations. Professional cultural evolutions or reformations in Italy have sparked in the field of nursing since 1992 and has resulted in major changes in the profession. Managing professional change is viewed as an essential component of health care system reform in several countries (Ayala, Fealy, Vanderstraeten, & Bracke, 2014). Patient safety (Affonso et al., 2007; Manojlovich, Barnsteiner, Bolton, Disch, & Saint, 2008) and the adoption of evidence-based practice, for example, have triggered changes that transformed the “culture” of how and by whom health care is delivered. These cultural shifts are underscored by reforms in health disciplines in Europe and are particularly evident in nursing within recent professional educational directives (Lahtinen, Leino-Kilpi, & Salminen, 2014).

In Italy, professional cultural reforms connote different perspectives among the health professions. For example, changes in the culture of Italian medicine focus on physicians becoming more flexible in their power and control of health care via collaboration and partnership with other health professions as well as their patients. In contrast, cultural reforms for Italian nursing are largely compelled through public laws, which are enacted by the Italian Ministry of Health. Professional development and university-based education are critical to establishing clinical competency, the foundation for professional autonomy and decision-making. Yet, these decisions largely reside in socio-political and governmental forces as described below.

The Decree of the Ministry of Health 739/1994 (called Professional Profile) (Destrebecq, Lusignani, & Terzoni, 2009; Stievano, De Marinis, Rocco, Russo, & Alvaro, 2012) provided the first recognition of professional autonomy for nurses in Italy (enacted in 1990). Other laws followed that officially ratified nursing professional autonomy (Sala & Manara, 1999) and established key competencies. A primary outcome of these efforts was critical academic nursing changes: a 3-year university degree in 2001 (Dante, Petrucci, & Lancia, 2013; Davies, 2008), masters’ degree in nursing science in 2004, and the first doctoral programs in nursing offered at four universities in Italy in 2006–2007.

The National Federation of IPASVI Colleges is a nonprofit body by public law established in 1954 to represent the nursing profession on a national basis. IPASVI coordinates Italy’s Provincial Colleges, which hold the registry of the nurses. It addresses two primary aims: the first is “protection of the person” which established the right of the individual, as sanctioned by the Italian Constitution, to receive health care services by qualified personnel who hold a specific fitness-to-practice title. The second aim, specific to registered nurses in Italy, sustains “professionalism” by (a) supporting equitable treatment, (b) ensuring that the deontological code is respected, (c) endorsing the professional cultural growth of registered nurses, and (d) institutionalizing good professional practice. IPASVI regulations were created to ensure maintenance of practice, competencies and compliance with their stated ethical code.

In Italy, the advancement of nursing scholarship catalyzed the profession’s cultural reform. This trans-formation is most evident in the higher status of the nursing profession country-wide. In 2010, IPASVI-Rome launched a Center of Excellence (CoE), first-of-its-kind, national infrastructure specifically designed to advance the field of nursing in Italy. IPASVI-CoE integrates education, research, and practice and is building a “culture of scholarship” (Boyer, 1990) to revitalize and elevate nursing as a health profession throughout the country. As part of its work, IPASVI-CoE seeks to identify and mitigate challenges that could potentially destabilize ongoing efforts to elevate the identity of nursing as a self-regulated health profession. Recognizing that there has been a lack of systematic studies to understand and document the impact educational and policy reforms have had on nurses and on current practice, we sought to explore nurses’ perceptions of their own professional growth and/or evolution as well as critical reforms that affected the culture of nursing as a health discipline.

## Method

### Design

We used a qualitative focus group design. Specifically, we leveraged the distinctive Italian style of focus group discussion (FGD), whereby metaphors are frequently used to communicate meaning and passion about a given topic (Carpenter, 2008; Sandelowski, 1998). Italians favor emotionally laden verbal and reciprocal interactions in conversation, which intertwine cognitive and psychological overtones and use rich Italian vernacular. Based on a prior patient safety study, in which a metaphor of “landmines in health care landscape” (Affonso et al., 2007) independently arose via group discussion, we learned that Italian nurses preferred using metaphors during group discussions to capture the complexities of their clinical experiences.

Metaphor is used as a primary vehicle to enhance understanding of the scientific and artistic approaches investigating a phenomenon (Kinn, Holgersen, Ekeland, & Davidson, 2013; Sharoff, 2013). Marshak (1993) noted that the use of metaphor as a form of symbolic, rather than a literal expression, has been shown to be an effective medium for describing an experience or a perception, which allows for “understanding and presenting ideas, insights and intuitions not always available to analytic reasoning and discourse” (p. 45). In other words, metaphors underscore depth of meaning and passion with topical content. This symbolism enhances interpretation and deeper understanding of sensitive and complex issues, including those presented in this article.

### Sampling

A convenience, purposive sample of 66 nurses (22 males and 44 females) enrolled in the study. Study participants included a cross-section of nurses between 22 and 65 years of age (mean of 42.1 years), with varying levels of education and clinical experience. Enrolled participants either had knowledge of the research topic or best represented the field (Elo et al., 2014). Focus groups were based on educational background or professional role/work setting of participants. They included (a) clinical hospital-based nurses (*n* = 10); (b) nurses in community practice (*n* = 9); (c) a mixed group of hospital and community nurses (*n* = 9); (d) designated “nurse scholars” (*n* = 6), including three deans of schools of nursing; (e) hospital nursing directors (*n* = 8); (f) doctoral nursing students (*n* = 8); (g) master’s nursing students (*n* = 7); and (h) baccalaureate nursing students (*n* = 9).

### Data Collection

FGDs were the primary method of data collection. Eight focus groups were convened in different regions of Italy with the majority held in Rome between December 2012 and July 2013. Study authors used a semi-structured focus group format. Prompt questions to stimulate group discussion were as follows:

- What is the meaning of the “culture of change” that pertains to clinical nursing practice during the past 10 years in Italy?
- What is the connection between the nursing cultural change and the advent of evidence-based practice in Italy?
- What are risks associated with nursing’s cultural change?
- What are the perceptions of professional nursing cultural change in Italy by patients?
- What (if any) is the contribution of IPASVI toward either positive or negative cultural change in nursing practice and research?
- How do other health professionals perceive nurses’ cultural change?
- What consequences are there for the future of the nursing profession in Italy if the current change did not happen?

Focus groups were 60 to 90 min in duration, facilitated by an Italian nurse investigator (fourth author), and conducted in Italian. Italian translators, fluent in English, transcribed the FGDs under the supervision of the first author. This facilitated and enabled full participation by all study authors, including those who did not speak Italian. Focus group sessions took place in a private conference room. Participants sat at a round table to maximize visibility and communication; group size ranged from 6 to 10 participants. At the outset, the group facilitator addressed group dynamics, including issues such as censoring and conformity. Undergraduate nursing students (three, one observer per focus group), trained as participant observers, took notes, paying particular attention to nonverbal behaviors and dynamics during group sessions. Transcription and content verification enabled (a) retrieval of key words and phrases, and (b) detection of nonverbal communication, gestures, and behaviors.

### Data Analysis

Focus groups data were transcribed verbatim within 2 days of the group session. Written transcripts of tape recordings created by Excel program were used as worksheets for coding. A coding team, composed of the fourth author and three trained undergraduate nursing students, analyzed the worksheets and identified commonly used words or phrases that were central to the group discussion. This in-depth analysis resulted in the development of a codebook. The fourth and the fifth authors of the study, assisted by a group of trained undergraduate nursing students, then developed categories, based on the codebook, which reflected topics, issues, and other concerns of importance to the group. Outcomes included phrases and descriptors representing categorical evaluations of the codebooks/worksheets.

A thematic and inductive content analytical approach (Elo & Kyngäs, 2008; Graneheim & Lundman, 2004; Hsieh & Shannon, 2005) helped to derive and interpret both manifest and latent content (Lipscomb, 2012) of the transcribed group discussions. We achieved saturation of the data through discussions and several meetings among the categorical team members, resulting in written summary notes. Study authors convened to generate themes from the codes and categories. As a result of a series of week-long face-to-face meetings and critical analyses of data sets, the team reached consensus on inductively derived themes and decided to use the semantics and vernacular elucidated by nurse participants in articulating the themes.

The research team adopted standards of rigor according to Lincoln and Guba (1985) and Guba and Lincoln (2005). We established dependability through coding, sub-categorizing, and categorizing (Bryman, 2008). Credibility was demonstrated through ongoing, interactive engagement in the data. Reflections and decision making emerged via investigative team discussions (face-to-face and by Skype) to increase credibility of the interpreted data. The attendance and observations of the three undergraduate nursing students in the focus groups helped the research team to better comprehend the weight of nonverbal behaviors of the participants. To ensure transferability, the researchers re-examined the database from their different point of views and cultures to delineate relationships among the various prompts for a broader scope and global comprehension of the contextualized information. A team of selected nurse participant representatives checked the research findings to ensure that they were true to nurse participants’ perceptions (Saldaña, 2013).

### Ethical Issues

Pertinent ethical committees in nursing schools involved in the study, where the different nurse participants gathered on a voluntary basis, approved the research and assured the study was conducted in an ethical manner. All participants signed a consent agreement signifying acceptance of the procedures involved in study, the focus groups in particular. Study authors provided written assurance to participants for protection in confidentiality of the data. The audio recordings from each session were transcribed and labeled according to a predetermined coding system to protect the identity of participants.

## Results

Six themes emerged that provide insights into nurses’ experiences with changes occurring in Italy during the past decade (2001–2011). Three of the six themes incorporated metaphors. “Vortex” described the turbulent changes and transitions associated with the cultural evolution of nursing as a health profession. “Leopard spots” represented the innate unchanging way of the old regime whereas “Deductive jungle” conveyed nurses’ individual struggles in clinical decision making throughout their professional experiences. Three other themes highlighted the complexity of Italian nurses’ experiences. These included *coraggio* (Italian semantics, meaning courage), signifying nurses’ resilience and fortitude to traverse negative forces; investment in nursing infrastructure development; and paradoxical dilemmas permeating changes in Italian nursing culture.

### The “Vortex” of Change in Nursing’s Cultural Evolution as a Heath Profession

Nurses described experiences of more gradual growth in their circumstances of change: “We are in an evolution, not a cultural revolution, of nursing whereby Italian nursing comes up to par with global nursing.” Nurses likened their clinical world during the past 10 years to a “sea of undercurrents” with several noting the tensions and turbulence coinciding with their professional development and other health care reform-related changes. Doctoral students and nursing directors alike used the “vortex” metaphor to describe the overwhelming sense of confusion they felt and experienced as well as what was perceived as a strong resistance to change.

Ongoing socioeconomic-political forces in Italy were thought to contribute to the persistent lack of autonomy among nurses: “Nurses are caught in and disappearing within a vortex of shifts in changes every day.” Another study participant noted that, “Nurses are overwhelmed by conflicts and autonomy issues, which are spiraling out of control in this time of our professional cultural evolution.” This descriptor was also used to depict generational tensions between more established nurses and those recently entering the profession; the incongruence between theory and practice; and, in clinical settings, which were said to be rooted in old ways amid a new cohort of nursing professionals striving for new standards of care.

Nurses also used the vortex metaphor to convey a sense of being overwhelmed by high patient workloads, which were made more complicated by sparse decision-making authority and a lack of autonomy to actively participate in formulating clinical care solutions:

Cultural evolution of nursing is a slow process; nurses going around and around in a vortex, overwhelmed by changes, discourse, less power and no solutions.

Nurses are in a vortex where the only objective is to come to the end of the shift and then come out from that vortex. But then you enter the vortex next time, when the nurse carries out a series of tasks, and you are cycling in the vortex once again. End of story.

Nurses described the evolutionary process, using terms such as searching for meaningfulness; striving to manage changes at all levels of physical, psychosocial, ethical and legal ramifications; hoping for new standards of practice congruent with international nursing protocols; and struggling with autonomy to evoke a new image of professional nursing in Italy. This is exemplified by several quotes of clinical nurses and nurse directors: “We are seeking nursing autonomy for a better and more meaningful image of Italian nursing.” “We need to have modern models to increase our motivation and better manage the changes upon us.” “We strive for professionalism; it’s beginning now in Italy. Otherwise, why be a nurse? The social context of nursing is important for our professional identity.” The complexities that underpin the vortex were also discussed by doctoral students as follows:

The decisional autonomy, the right way to communicate, the process of decision-making, the right way to practice is to be studied by us [nurses]. But this autonomy is a decision of that very moment and you still have to manage the patient. I have to speak with him/her [the patient] but I have to comprehend his/her deep reasons . . . . The practice is changing. I can be very skillful but I have to be ready to greet this challenge and, in this way, my mind is changing too.

### “Leopard Spots” Represent the Innate, Unchanging Way of the Old Regime

Several groups discussed the forces that underscore resistance to change internally (within the nursing profession) and externally (from other health professions). The complex nature of these forces were reflected by the Italian nurses’ reference to the “leopard spot” metaphor. “Leopard spots” are highly visible: “They are innate to who the leopard is, and cannot be removed.” In this context, nurse participants discussed how most nurses are entrenched in who they are, how they want to be perceived, and how they practice their profession. Head nurses, physicians and hospital units, and even the universities were singled out as having “leopard spots” by the nursing directors’ group as illustrated by the following quotes: “Leopard spots don’t change; some nurses, even head nurses, and physicians can’t change, don’t want change and deter change. This impacts multiple areas, including education of the young.”

Other quotes included the following: “Leopard spots are in the territory of evolutionary changes. Leopard spots are even in the higher positions in nursing, especially head nurses of old models.” “Completely different mindset creates resistance, conflict and stress. Older generation [of nurses] may not see value of ethical and psychological preparation.” “Even today undergraduates are coming out of universities that have the famous ‘leopard spot’; their educational teaching drives them to a task-oriented work and hence [these new nurses] don’t have any enthusiasm.” Doctoral students elaborated on other dimensions of the “leopard spot” metaphor: “It’s impossible to regress; we must go forward but the gap between older and younger nurses gets deeper, so going forward is harder.”

Study participants noted that the changes are destabilizing and creating turmoil individually and collectively: “In ten years, there have been many changes but these sudden changes created great confusion.” One of the participants noted that “Old systems are [still] dominant in Italy. It’s difficult to make change on the units. Nurses continue old ways; professionalism [is] still lacking and the focus is on technical tasks.” Given the role of head nurses in effecting policy change and assuring compliance, the “leopard spot” metaphor allowed a more nonthreatening way to identify those who were resistant to change, and how to build buy-in and support for new policy and practices.

### The “Deductive Jungle”

Building on the interplay of the “vortex” and “leopard spot” metaphors, Italian nurses also referenced and elaborated on the metaphor of “deductive jungle” used to convey nurses’ individual struggles in clinical decision making throughout their professional experiences:

Nurses are living in a deductive jungle; critical aspects of the profession are variable, [such as curricula] . . . or developmental, [such as knowledge of practice guidelines]. We weren’t taught reflective clinical decision-making so we have to rely on our own logical thinking.

Master’s students emphasized that “There is a huge variability in educational curricula, because we have no common curricula for nurses in Italy, which puts us in a deductive jungle in practice. Different content standards create confusion.” Clinical nurse participants stressed the need for guidance on how to navigate the “deductive jungle” to practice competently. Barriers, dangers, risks, and lack of solutions to persistent clinical problems were cited as factors that powered the “deductive jungle.” Variations of this theme ran across the eight focus groups. Master’s students also made reference to head nurses as part of the “deductive jungle”: “Today the head nurses’ job is very bureaucratic and their competencies are not updated. This continues the deductive jungle. We are doing very little to control classes of head nurses.”

### “Coraggio,” Signifying Nurses’ Resilience and Fortitude to Traverse Negative Forces

“Coraggio” signifies the depth of psychological resilience necessary to traverse negative forces encountered. Focus groups utilized many descriptors to characterize the range of emotional turmoil and, at times, instability and fear experienced by and perceived by nursing professionals.

Study group participants stressed that nurses needed courage to endure changes, make decisions, and feel a sense of value and worth. Used by several groups to communicate how courage underpins change, the Italian expression of “coraggio” is deeply valued and practiced by nurses: It is an ethical value often associated with the exposition of social phenomena in Italy. Coraggio connotes the complexities and intricacies that underscore the difficulties a person experiences when he or she finds himself or herself in hard and threatening situations. Clinical nurses’ focus group expressed that:

Nurses have lots of fears; fear of changes; fear of losing autonomy; fear we are not competent . . . If a ward, department doesn’t work, it is [the] responsibility of the nurse manager to raise problems and try to solve them, through life-long learning. We need courage to do change. We need ways to be courageous.

Clinical nurses also emphasized that because health care is very difficult to change in Italy, coraggio had to be not only at the individual nurse level, but an ethical commitment for all health professionals to comply with the deepest values of their conscience and their professional identity:

Courage is not merely to help us in difficult situations of our practice. It also has to be in the mindset or conscience of the nurse. Since coraggio is [one of] our deep Italian values, it has to be in our ethical code in our professional identity.

The doctoral student focus group emphasized a unique dimension of coraggio, namely, that of social change and public policy changes when they commented on the need for courage to push policy forward and sustain policy for professional development. They noted, the “Reason for change is to emancipate nurses from the system. But do we have the courage to do so?”

Master’s students reflected on coraggio as an Italian pathway:

Change is very difficult, because even if I have got skills and I’m aware of it, I have to put myself under discussion to change practice and I have to go against the team. For me, this is the pivotal passage. Coraggio is the way.

Similarly, the nurse directors’ focus group brought forth another dimension of coraggio, that of advocating for patients and families by making patients first in patient care:

In reality, one definition of centrality of the patient in the nursing care talks about a shared responsibility between health professionals and patient. We don’t apply the centrality of the patient care and we should be more congruent with this kind of patient care innovation!

Coraggio was also discussed relative to its dimension of economics that drive social-public systems change. Specifically, study participants recommended that IPASVI-CoE (a) finance the continuous development of nursing, (b) sustain reforms to secure nursing as a health profession overtime, and (c) expand the CoE’s role:

We appreciate two key elements; first is the courage to set up a Center of Excellence, knowing that many nurses lack awareness of the true potentialities of nursing scholarship in Italy. Second, is the economic courage to sustain changes for nursing reforms.

“We have to do something with the means we have, with our IPASVI, with our professional associations, with our Regulatory Boards, with the transformation of our image. The substance is present, but we have to give it visibility,” said a study participant.

### Investment in Nursing Infrastructure Development

All focus groups, the nurse directors in particular, expressed the need for strong and enduring investments, including increasing intellectual capacity, support-based sources, and stable fiscal resources. Several building blocks were found to be critical to cementing nursing infrastructure in Italy. These included (a) critical processes (to build, manage, and sustain changes), (b) resources (including physical and intellectual capacity and funding), (c) multi-level supports including intra and intercollaborations (involving other disciplines), (d) cross-sector partnerships including the Italian Ministries of Health and Education, and (e) active community supports.

Nurse directors emphasized the need for new strategies and systems: “Many nurses perceive no supportive environment to cope with changes in nursing. We need strategies and new systems to manage change in nursing.” Nurse scholars added, “Economic, societal context of change means we need new alliances, partnerships and stakeholders.” Nurses from all groups cited how the IPASVI-CoE is the only current infrastructure operating to advance nursing in Italy. They expressed awareness and also concern that other resources were urgently needed to sustain the IPASVI-CoE’s infrastructure: “IPASVI-CoE is the rock of salvation for nurses in Italy; it [is] the major support system to promote professionalism of nursing in Italy.” Another participant noted that “IPASVI-CoE gives opportunity for us to reflect practice, talk of changes, and have a path to a better future in nursing.” Moreover, nurse directors added,

We have no integrated system for nursing education, practice and research for us to come together in shared vision and advance nursing in the country. Only one system has possibilities for us, IPASVI-CoE. But one structure is not enough.

Study participants also noted that “IPASVI has a pivotal role in promoting nursing as an ethical profession, demonstrating value of the cultural evolution processes of nursing and portrays a normative view point of nursing as equally important to other health professions in Italy.” Some nurse directors, however, declared that “IPASVI has to be present effectively in all regions of Italy and not only at ‘leopard spot.’” Different visions of the various IPASVI Regulatory Boards were noted as an obstacle to achieving a shared vision that could guide the profession. Nurses across the majority of groups also discussed the importance of life-long learning, with the IPASVI-CoE being cited as one of the leadership institutions that provides opportunities for professional development via new educational programs: “Life-long learning is important for the contemporary nurse in Italy. Basic education is not enough. Through IPASVI, we have easy access to existing programs and opportunities for new programs that bring global nursing models to us.”

### Paradoxical Dilemmas Permeating Changes in Italian Nursing

Contradictions were revealed via nurses’ stories that pointed to paradoxes of practice. Four paradoxes were salient throughout group discussions. The first paradox pertained to the contradiction that amid the momentum and advances in nursing education, there is the reality of what was described as a lack of “innovation” throughout the Italian health system: “The change that has occurred at the educational level and at the health professional level, has not been realized at the practical level; it never ever passed into practice to make any difference.”

The second paradox related to the contradiction that whereas unions organize nurses and unite them through contracts that include more benefits and protections, they continue to sanction old roles of the nurse not recognizing advanced nursing practice roles as valid for union contracts in Italy.

Unfortunately, we, in Italy, have unions that are not ready to value nursing competencies. Because the unions fear that nurses with competencies can create difficulties for nurses who don’t have up-to-date skillsets . . . We have this big problem. For example, possibilities exist for a nurse to have a managerial career. But if a nurse desires to pursue a clinical career, such as in advanced nursing practice, this is not recognized by availability in working contracts.

A third paradox (highlighted by master students) was the contradiction that a real sense of nursing was lacking in the current nursing science in Italy. In fact, nursing education was not completely autonomous in the Italian universities, as expressed below.

Nursing science is under the umbrella of the faculty of medicine and this has hindered, ghettoized and cordoned off the development and evolution of nursing. Why? It’s sufficient to read the didactic set of rules, it’s full of biomedicine. We are educated health professionals who are doctors in miniature, with small skillset. I want to say there’s a contradiction that a real sense of nursing is lacking in our nursing science.

The fourth paradox was the contradiction of distinctively different standards of quality of work offered by private institutions versus public institutions even if nurses were accountable to providing parity in patient care.

Private institution versus public institution is of another culture. Public atmosphere is more relaxed; private more controlled. So the care is different. How are we to give quality standards of care? It is an ethical dilemma for us nurses, especially when we know models of global nursing standards.

## Discussion and Conclusion

Study authors delineated the characteristics, meanings, and challenges that Italian nurses experienced in their changing culture as a health profession. The “vortex” metaphor disclosed powerful images of conflict and struggle as encountered by nurses, particularly among clinical nurses at the hospital level, and by nurse leaders. A host of legislative changes established university-level (baccalaureate) education as the minimum entry requirement for nursing. Other statutory changes solidified the elevation of nursing as a credentialed and recognized autonomous health profession in Italy, which gave way to increased professional development opportunities. Although these changes were largely seen as progressive (Barazzetti, Radaelli, & Sala, 2007; Sala & Manara, 1999; Sala & Usai, 1997), Italian nurses perceived them to be controversial. Because multitude of changes occurred in a relatively short period of time (1992–2006), they incurred instability and sometimes chaos in patient care settings.

Many clinical settings were still rooted in archaic mind-sets that relied on old models of care. Necessary changes had not been fully embraced in health care organizations, and as a result, with little or no institutional buy-in or designated resources, there had not been widespread improvements in the quality of patient care. Nurses still suffered from a lack of recognition and low levels of autonomy and responsibility. In many clinical settings, rigid administrative hierarchies prevailed (Camerino, Conway, et al., 2008; Camerino, Estryn-Béhar, et al., 2008; Cortese, 2007; Destrebecq, Terzoni, Colosio, Neri, & Brambilla, 2009). The lack of professional autonomy (Öhlén & Segesten, 1998; ten Hoeve, Jansen, G., & Roodbol, 2013) was associated with limitations in independent clinical decision-making processes (Traynor, Boland, & Buus, 2010a, 2010b). Few opportunities to obtain advanced practice education (Benner, 1984; Daly & Carnwell, 2003) and minimal access to managerial roles that permitted engagement in policy making were known to contribute to this problem.

Use of the “leopard spots” metaphor to illuminate the strength of old regimes (still prevalent within the Italian health system) revealed the true nature of “obstinate, resistive” forces to changes in health care system reform. Many nurses noted that the “hard core of the Italian health system difficult to break” and the persistent “medical umbrella” were obstacles to advancing not only nursing but also other health professions. The medical and biomedical culture is still strong in Italy, especially in the center-south of the country and in rural areas as it is in most of the Southern European countries (Palese et al., 2013). Although partial decline has occurred over the past two decades (Tousijn, 2002) and some aspects of innovation are visible (France & Taroni, 2005), the Italian “old regime” remains stalwart.

Nurses cited the need to change the model of health care delivery in Italy. Nurses believed that for their profession to advance, a critical change had to occur. Namely, Italy had to move from the current pivotal role of hospital-based medically dominated care to a more regional-territorial approach that enabled interprofessional models of collaborative care (Price, McGillis Hall, Angus, & Peter, 2013).

The finding that Italian nurses described their professional evolution as akin to being in a “deductive jungle” warranted further study. This metaphor could lend insights into understanding health professionalism. The Italian emphasis on how variability in nursing curricula and standards of practice fueled the deductive jungle might inform teams’ collaborative initiatives specific to decision making that involve teams composed of clinical nurses, students, and nurse educators. Preliminary evidence, however, told us that reflective clinical decision-making was a means to cope with the “deductive jungle” and the IPASVI-CoE had to use it as a source of educational training, with a particular focus on skillset development in clinical decision-making.

Coraggio was communicated to underscore the importance of nurses’ psychological well-being when enduring the difficulties associated with change (Kidder, 2005). It also reflected the absolute will and sheer determination of nurses to conquer obstacles and be resilient and courageous in the face of challenges. Although emotional tensions, particularly anxiety and fears relative to change, have been reported in the literature (Jensen & Lidell, 2009; Khalili, Hall, & DeLuca, 2014; Lachman, 2007; Lindh, Barbosa da Silva, Berg, & Severinsson, 2010), Italian nurses brought new insights regarding how emotional turmoil experienced in the most difficult of times related to their Italian value of coraggio was used to advance professional development and innovative patient care.

In conjunction with the need for courage, we found that younger Italian nurses were particularly concerned about maintaining a firm focus on the relational aspects of patient care. Patient-centered care as a core in health care quality is well known and valued globally (Kunyk & Austin, 2012). An exemplar is demonstrated in Latin countries where the juxtaposition of relational dimensions to quality care is manifested through expressive communicative styles that characterize strong relationships between patients and health care teams (Manara, Villa, & Moranda, 2014). In appreciation of this context, Italian nurses expressed concern over the lack of attention to the nurse–patient relationship because of clinical variables such as heavy workloads, scarcity of health care support staff, and the burden of a “fixed list of work tasks.”

Italian nurses from both the younger and older generations expressed a mixture of confusion and frustration about how to manage the implications of cultural changes in both practice and education. Nurses communicated the need for help in resolving “paradoxical dilemmas” via stronger advocacy and support. A specific challenge that was addressed was the current limitation of advanced practice roles for nurses in Italy that stunted nursing as a career. Accordingly, study authors recommend that IPASVI-CoE takes the lead in advocating for change and additional nursing reforms at multiple levels including, but not limited to, the Italian Ministry of Health, the Unions and the National Board of Physicians. Nurse participants noted how difficult it was to build a career as clinical nurse leaders because they were likely to be penalized in their actual work, as sometimes seen when nurse administrators in powerful positions engaged in clinical “bullying” in the workplace (Magnavita & Heponiemi, 2011).

Nurse participants overwhelmingly affirmed that additional work was needed and that IPASVI-CoE was ideally suited to monitor the change process, provide support to nurses at all levels (students, clinical practice, nursing directors), produce guidelines and recommendations, and make resources accessible through ongoing policy imperatives. Although it was clear that IPASVI-CoE was pivotal to advocating changes and advancing Italian nursing, concern existed whether a singular nursing infrastructure was sufficient to maintain and sustain the changes in nursing practice and education that were needed in Italy. Study participants suggested there might be a need to create new alliances and stakeholders to sustain IPASVI-CoE, with some suggesting that an infrastructure separate and apart from IPASVI be established to support nursing research as part of the Italian Ministry of Health. Moreover, strong investment in clinical practice via other innovative programs that focus on “life-long learning” (cited as an important goal by nurse participants) and evidence-based practice was noted by group participants.

Noteworthy, partnerships between IPASVI, the Italian Ministries of Health and that of Education, and other key nursing organizations were discussed as future possibilities based on strong relational commitments. Study participants also recommended consideration of strategies to strengthen the smaller, localized branches of IPASVI at the provincial level throughout Italy. The influence of a national professional organization such as IPASVI to break down barriers in the Italian health care system that impede progress was viewed as essential to building on current nursing reforms and sanctioning achievements made in clinical practice and education to date. In conclusion, this study presented valuable information for program development that can continue to build and support the practice, education, and research directives of Italian nurses. Specifically, the three metaphors can be used to inform and conceptually frame more conventional, rigorous studies regarding the evolution of Italian nursing.

## Limitations and Strengths

The questions used to stimulate group discussion were likely too numerous; this precluded a fuller in-depth discussion, which was the goal of conducting focus groups. The sample limited comparisons or contrasts of nurses’ perceptions because most of the participants were largely urban nurses from Rome and Genoa. This limitation, however, enabled two strengths of the study. The sample included a wide range of nurses from the students of undergraduate, master’s, and doctoral programs to hospital-based and community nurses, nurse directors, and nurse educators/scholars. The inclusion of three generational levels of nurses (ranging from young students, novices, experienced clinical nurses, and seasoned head nurses) provided a broad lens through which to examine the meaning of change. Second, the study was the first to describe Italian nurses’ perceptions of cultural changes in their professional evolution.

## Study Implications

The research team made a contribution to the field by making salient the significance of metaphors that enrich group dynamics in qualitative research. The vivid use of metaphors and choice of particular semantics helped to more fully communicate the evolutionary process of the field of nursing in Italy, including its different levels of meanings, passion, and concomitant needs. Although study findings were specific to nursing, the six themes, particularly the metaphors, were relevant to implicating evolutionary changes within other disciplines undergoing cultural shifts in their practice and identity. Furthermore, these metaphors shed insights into understanding system changes, such as that of the Italian health care system. In this regard, study findings had implications for the Italian Ministry of Health.

As follow-up to this study, IPASVI-CoE leaders presented these findings to the Minister of Health and her staff. Favorable outcomes of their dialogues and debates included subsequent meetings on possible next steps to plan and implement collaborative and interdisciplinary models of care that has yet to become vibrant in Italy. In this regard, IPASVI’s annual national conferences and biennial international congresses will feature the metaphors, specific to their implications for innovations in patient care. In terms of further studies, a call for proposals (by IPASVI-CoE) for projects on how the nursing metaphors can inform clinical practice and health care in Italy will be made.
